# A mixed methods evaluation of the feasibility, acceptability, and impact of a pilot project ECHO for community health workers (CHWs)

**DOI:** 10.1186/s40814-020-00678-y

**Published:** 2020-09-18

**Authors:** April Joy Damian, Sarafina Robinson, Faaiza Manzoor, Mandy Lamb, Adriana Rojas, Ariel Porto, Daren Anderson

**Affiliations:** 1 Weitzman Institute, 19 Grand Street, Middletown, CT 06457 USA; 2 Johns Hopkins Bloomberg School of Public Health, Baltimore, USA

**Keywords:** Community health workers, Project ECHO, Workforce training, Health equity

## Abstract

**Background:**

Despite the positive effects of community health workers (CHWs) on addressing social determinants of health, improving patient health outcomes, and decreasing overall healthcare costs, there is a lack of standardization in training and certifying this workforce, resulting in different approaches to integrating this role into medical home models. The purpose of the current study is to evaluate the application of Project ECHO (Extension for Community Healthcare Outcomes) in enhancing CHWs’ capacity to address health and social issues of vulnerable populations.

**Methods:**

An explanatory sequential mixed methods design was applied in which all participants (*N* = 49) completed pre (January 2019) and post (July 2019) quantitative online surveys measuring changes in self-efficacy, behavior change intent, and knowledge. Virtual focus groups were conducted with a subset of participants (*n* = 20) in July 2019 to assess the feasibility, acceptability, and impact of Project ECHO.

**Results:**

There was a statistically significant difference of + 0.453 in the composite self-efficacy mean score pre- to post-series. For every 1 additional Project ECHO CHW session attended, there was a .05 improvement in participants’ self-efficacy to perform CHW-related job duties and address social determinants of health (SDOH). Four major themes emerged from the qualitative focus group data: value in learning from other participants’ caseloads, CHW-care team integration, availability of training and resources, and shared decision-making with patients.

**Conclusions:**

This evaluation suggests that ECHO is a viable means of increasing access to training resources for CHWs. Future studies on the ECHO model as a means of educating and broadening implementation of CHWs are warranted. Programs such as Project ECHO can support CHWs by providing continuing education opportunities, as well as standardizing training content across large geographic areas.

## Key messages regarding feasibility

- This evaluation suggests that Project ECHO can be a feasible and acceptable tool to provide continuing education and training for community health workers (CHWs). Participants shared how certain skills and knowledge obtained from ECHO CHW could be deployed in their respective communities and healthcare settings. Knowledge and self-efficacy gains in didactic areas such as integrating trauma-informed care, enacting motivational interviewing skills, and changing patients’ health behaviors indicated its impact.
- The participants’ positive regard of the curriculum topics, didactic presentations, and case recommendations demonstrated acceptability. In acknowledging that the video conferencing environment was conducive to their learning needs, ECHO CHW participants provided context for the initiative’s overall feasibility.
- The current study’s feasibility findings suggest that Project ECHO model can play an important role in supporting and providing CHWs with continuing education to help address challenges faced by patients beyond the traditional domain of healthcare provision. Programs like ours that train and support CHWs are increasingly important as health systems, prompted in large measure by changes in payment models, seek new strategies to improve quality and value.

## Introduction and background

Health initiatives that target social determinants of health (SDOH) underscore the notion that a patient’s environment influences their overall health and well-being. Quality improvement efforts in healthcare have traditionally focused on ameliorating poor health indicators within clinical practice [1]. Although this approach may lead to improvements in some health outcomes, it often fails to account for the significant influence of health-related social factors such as housing, access to food, transportation, and employment.

Interventions that address SDOH are based on the emerging consensus that improved health outcomes cannot be achieved by the healthcare sector alone. Rather, collaboration among healthcare providers, patients, and patients’ natural supports (e.g., family, friends, community members) is necessary to improve health and promote wellness. Federal initiatives, such as *Healthy People 2020*, have established national objectives to create environments that promote good health for all [2]. Initiatives like these identify the most pressing preventable threats to health and establish indicators to diminish these threats. These initiatives target local communities and emphasize whole-person-centered healthcare systems supported by programs such as Community-Clinical Integration Programs and Patient-Centered Medical Homes [3, 4]. Efforts to improve healthcare access, lower healthcare costs, and improve population health are at the crux of these initiatives and underscore the role SDOH play in population health and well-being.

One workforce functioning at the intersection of healthcare and community is community health workers (CHWs). The American Public Health Association defines a CHW as the following:

A frontline public health worker who is a trusted member of and/or has an unusually close understanding of the community served. This trusting relationship enables the worker to serve as a liaison/link/intermediary between health/social services and the community to facilitate access to services and improve the quality and cultural competence of service delivery [5].

Fostering trust between community residents and healthcare systems is not a new concept. For more than 50 years, CHWs have worked to address healthcare concerns, expand access to preventive health services, and mitigate health disparities in historically vulnerable and medically underserved communities [6]. CHWs bridge language, literacy, and cultural meanings for people and communities while promoting health education, care coordination, and linkages to resources [7–9]. By assuming a broad range of responsibilities, CHWs bridge the gap between primary care provision and well-being outside the clinical setting, particularly among patients most in need. Several studies have demonstrated reductions in care utilization and more equitable access to curative services for vulnerable patients managing chronic health conditions when CHWs were included in the patient’s care team ([10–13]; Moffett M, Kaufman A, & Bazemore, 2019 [14];). One randomized control trial of CHWs supporting low-income patients with multiple chronic conditions demonstrated improvements in health outcomes, mental health, and reductions in hospitalizations [7]. Similarly, a systematic review revealed that CHW interventions can significantly reduce emergency room visits, hospitalizations, and urgent care visits among patients served in various healthcare settings [12].

Despite the positive effects of CHWs on patient health outcomes and overall healthcare costs, there is a lack of standardization in training and certifying this workforce, resulting in different approaches to integrating this role into medical home models. A national survey of the CHW workforce revealed variations in role definition and training criteria to support their professional development [12]. Online and in-person training resources for CHWs also vary considerably in content, quality, and access [15–17].

One promising approach to addressing this gap is Project Extension for Community Healthcare Outcomes (ECHO). Project ECHO is a novel educational intervention that uses case-based learning and video conferencing to connect experts in a particular discipline with frontline healthcare workers. Since 2003, ECHO has been used to connect primary care providers in medically underserved environments with ECHO specialists in order to supplement knowledge and enhance self-efficacy regarding treatment of specific diseases [18]. While there is emerging evidence that Project ECHO has a positive impact on provider behavior and self-efficacy, less is known about its utility as a tool to support non-medical provider members of the healthcare team such as CHWs. Few studies [17, 19] to date have evaluated the use of the ECHO model for CHWs. Moreover, prior applications of ECHO to educate CHWs focused on specific health conditions or diseases and were often designed for CHWs within narrow geographic regions. In this paper, we examine the feasibility, acceptability, and impact of a national Project ECHO training program created specifically for CHWs to enhance their competency and improve their ability to address patients’ SDOH. Prior implementation research related to Project ECHO has identified feasibility and acceptability as key components to ensuring implementation success [20], yet it is unknown to what extent CHWs will choose to participate in and have their learning needs met by a national ECHO with a broader curriculum. In addition to feasibility and acceptability, we also assess impact so as to understand immediate outcomes related to the intervention. To our knowledge, this is the first mixed methods evaluation of a national, innovative application of ECHO to train CHWs in addressing the health and social needs of their respective patient populations.

## Methods

### Project ECHO CHW intervention and sample

In an effort to support standardization and sustainability of training for CHWs, and with funding from the Center for Medicare and Medicaid Innovation State Innovation Model (SIM), the Weitzman Institute, a research organization embedded in a Federally Qualified Health Center (FQHC), partnered with the Penn Center for CHWs to pilot the implementation of Project ECHO for community health workers (ECHO CHW). Project ECHO CHW was a 6-month virtual, case-based learning initiative designed to enhance participants’ capacity to address health issues for vulnerable populations typically served by CHWs.

This ECHO series connected CHWs and those in similar roles, including hospital-based patient care coordinators, case managers, and patient advocates, with faculty mentors from the Penn Center for CHWs and Community Health Center, Inc. While specifically designed for CHWs, Project ECHO CHW program implementation staff permitted healthcare workers who facilitated healthcare and social service system navigation to engage in the series alongside their CHW peers. This enabled interdisciplinary dialogue among healthcare workers serving in similar capacities to the traditional CHW. The initiative had four distinct, but related goals: (1) to enhance participants’ knowledge of general CHW core competencies, (2) to augment participants’ capacity to address SDOH, (3) to assist participants’ in their ability to work alongside patients in the attainment of specified health goals, and (4) to build a national learning community where CHWs can receive support in working with their challenging patient cases, share resources with other participants regionally and nationally, and learn from an expert faculty panel.

### Faculty and curriculum

Faculty members, including one senior CHW, an assistant director of training for CHWs, a patient navigator, a program manager, and a nurse manager, were selected based on their expertise in the areas of case management, clinical care, community referral processes, and training CHWs. Learning sessions were 90 min and held twice monthly by video conference from January 2019 through July 2019. Faculty used approximately 30 min to present didactic lectures on curriculum topics such as qualitative interviewing, trauma-informed care, and connecting patients to community resources. The list of didactic topics covered during the ECHO CHW intervention is included in Appendix B. The remaining hour was devoted to case-based learning, which involved participants presenting details about the biopsychosocial challenges of patients they were supporting, and faculty leading a discussion and sharing recommendations for each particular case.

### Participant recruitment

The intervention was open to CHWs nationwide. Recruitment was conducted via email utilizing a national list of contacts primarily from FQHCs and other primary care practices and word of mouth within Connecticut. A total of 120 participants from 33 organizations across 19 states and the District of Columbia enrolled in the program. All participants were over 18 years of age and English speaking. The Institutional Review Board at the Community Health Center, Inc. approved all study procedures.

### Pre-post design

Pre- and post-surveys were developed through a collaborative, iterative approach between the research staff and program implementation team. Research and program staff developed a set of questions to gauge participants’ confidence to deploy skills related to certain didactic topics outlined in the initiative’s curriculum (*e.g., How confident are you that you can recognize a patient’s motivation to adopt a particular health behavior? How confident are you that you can connect a patient with community resources that meet that person’s needs?*). The survey was a 35-question instrument that captured participants’ roles, years of experience, prior training, age, race/ethnicity, gender identity, and responses to self-efficacy questions (20 items) aligned with 8 of the 12 ECHO CHW didactic topics. Participants completed the Participant Enrollment Form (baseline, pre-survey) prior to joining any of the 12 scheduled ECHO CHW sessions. The link to the post-series survey included the same set of self-efficacy questions (20 items) and was sent via email following the last ECHO CHW session to everyone who completed a Participant Enrollment Form and participated in at least one ECHO session. For data quality and management purposes, ECHO CHW participants were assigned a unique participant code at the beginning of the learning series. Participants input their assigned participant code to access the baseline and post-series surveys, which enabled us to match pre- and post-surveys for data analysis. Additionally, a mid-series survey assessed participants’ satisfaction with various elements of the ECHO program in addition to self-reported behavior changes as a result of engagement in the intervention.

### Semi-structured focus groups

Two separate, 1-h focus groups with ECHO CHW participants were conducted at the conclusion of the program. The focus group questions sought to determine participants’ perceptions about the feasibility, acceptability, and impact of the program. A total of twenty participants took part in both focus groups. The semi-structured interview guide is included in Appendix C.

### Measurement strategy

This evaluation applied a sequential explanatory mixed methods design [21] in which open-ended focus group questions are used to provide explanation and context for survey responses (Damian, Gallo, Leaf & Mendelson, [22]). More specifically, the five parts of the focus group questions (Appendix C) contextualized the survey questions, which also primarily focused on examining content, attendance, resources, engagement and application, and support and relationships from the participants’ perspectives. Additionally, this evaluation’s methodology was guided by Moore’s Model of Outcomes Assessment framework, which has been applied to a range of other ECHO clinics (Zhou, Crawford, Serhal, Kurdyak & Sockalingam, [23]). The quantitative survey questions, in addition to semi-structured interview questions, align with levels 1 through 4 of Moore’s assessment framework, which is referenced in Appendix A.

#### Moore’s model of outcomes assessment level 1: participation

The Project ECHO program coordinator maintained program attendance records for the entire duration of the intervention through Zoom video conference reporting. Attendance confirmation emails were also sent immediately following each session due to limitations in Zoom capturing participants that join from the same computer or via telephone.

#### Moore’s model of outcomes assessment level 2: satisfaction

A mid-series survey was administered in April 2019 to assess participants’ satisfaction with and acceptability of this ECHO program. The survey included 11 satisfaction-related items on a 5-point Likert scale (1 = very dissatisfied and 5 = very Satisfied) and 6 items on a 5-point Likert scale (1 = strongly disagree and 5 = strongly agree) regarding the extent to which Project ECHO met participants’ learning needs. Participants also provided open-ended feedback regarding their overall satisfaction with the session’s didactic lectures, case presentations, and faculty recommendations.

#### Moore’s model of outcomes assessment level 3A: knowledge

The ECHO CHW faculty developed two to three multiple choice knowledge poll questions specific to each didactic session. These questions were delivered through a polling feature on the video conferencing platform at the beginning (pre-) and end (post-) of didactic sessions 5 through 12.

#### Moore’s model of outcomes assessment level 3B: self-efficacy

The 20-item self-efficacy survey measured participants’ belief in their ability to perform core competencies related to the didactic sessions using a 4-point Likert scale (1 = not at all confident, 4 = very confident). Higher mean scores indicated greater degree of confidence in participants’ ability to perform duties related to the CHW core competencies.

#### Moore’s model of outcomes assessment level 4: competence (behavior change intent)

Behavioral change refers to one’s attitude about the likelihood to engage in a behavior at a specific time and place. The mid-series survey included three qualitative questions that assessed participants’ intent to enact behaviors aligned with the case-based learning and didactics they had been exposed to up to the midway point in the entire series (*e.g., Is there anything you plan to do differently in your professional role based on knowledge you gained from Project ECHO CHW? Please describe how you plan to apply what you learned.*). Semi-structured focus group interviews conducted at the conclusion of the intervention and described in greater detail below included two qualitative questions regarding participants’ intent to change behavior based on lessons learned from the intervention.

#### Other covariates

Research staff also collected information about sociodemographic characteristics such as gender (male, female, not specified), professional role (community health worker, care coordinator, case manager, patient navigator), years of experience in role (< 1 year, 1–4 years, 5–9 years, 10–15 years, > 15 years), and prior training in CHW core competencies (yes/no).

### Analysis strategy

Descriptive and inferential statistical analyses of quantitative survey data were performed in IBM SPSS 22 and STATA 15. To measure internal consistency, an inter-item correlation was performed on the 20-item self-efficacy pre-post survey. The alpha coefficient for the twenty items was 0.93, suggesting that the survey had relatively high internal consistency. The alpha coefficients for subscales within the survey pertaining to the eight of twelve total didactic topics, however, varied. The separate subscales reflected eight of the twelve didactic topics outlined in the ECHO CHW curriculum and were comprised of two to three items each. The trauma-informed care subscale consisted of 3 items (α = 0.77), the mental health subscale consisted of 2 items (α = 0.81), the health behaviors subscale consisted of 3 items (α = 0.90), the re-engaging patients subscale consisted of 2 items (α = 0.27), the conflict resolution subscale consisted of 3 items (α = 0.73), the confidentiality subscale consisted of 2 items (α = 0.40), and the ending the patient relationship subscale consisted of 2 items (α = 0.92). The survey only contained one question on motivational interviewing. A total of 119 participants enrolled to participate in ECHO CHW and responded to pre-survey measuring self-efficacy. Five of these participants notified the program staff of their desire to discontinue their participation prior to the end of the learning series, and eight additional participants registered, however, never attended any of the sessions. Fifty-one participants completed the post-survey at the conclusion of the series, which resulted in a 48% response rate. As such, a latent change score model was used to calculate the extent to which changes from pre to post were significant when accounting for missing post-survey data due to pre-post survey completion attrition. The latent change score model created true change values in self-efficacy without ignoring missing data at the post-series marker. All descriptive and inferential tests of survey data were performed in STATA 15.

Open-ended survey responses and focus group data were analyzed qualitatively. Open-ended survey feedback was considered in the context of this evaluation’s aims and analyzed accordingly. Focus group data were transcribed from a video recording, de-identified for confidentiality, and reviewed independently by two trained coders from the research team. The research team read through each transcript three times, coding key statements. The iterative coding process that involved clustering of keywords was followed by thematic analysis, whereby a search for themes that emerged in the dataset occurred. Thematic analysis involves the identification of themes through careful reading and re-reading of the data [24]. A third trained coder from the research team was included to assist with developing consensus around themes and address any disagreements between the two initial coders. NVivo 12 Plus was used to organize and analyze focus group data.

## Results

### Sample characteristics

Table 1 shows the demographics of the analytic sample (*N* = 51). While about half of the participants (49%) identified as CHWs, a third of participants (33.3%) selected “Other (not listed),” indicating that they either held a different title but engaged in similar roles (e.g., recovery navigator), or supervised CHWs (e.g., directors, program managers). A small proportion (18%) of participants had less than a year of experience in their current role. Most participants were female (94%) and had prior training in CHW core competencies (55%).

**Table 1:** Demographics of analytical sample in Project ECHO community health worker training (*N* = 51)

Characteristics	Total ***N*** (%)
Professional role	
Community health worker	25 (49.0)
Case coordinator	3 (5.9)
Case manager	4 (7.8)
Patient advocate	2 (3.9)
Other (not listed)	17 (33.3)
Gender	
Female	48 (94.1)
Male	3 (5.9)
Years of experience in role	
Less than a year	9 (17.6)
1–4 years	25 (49.0)
5–9 years	7 (13.7)
10–15 years	3 (5.9)
More than 15 years	6 (11.8)
Not specified	1 (2.0)
Participated in any prior training related to CHW core competencies	
Yes	28 (54.9)
No	23 (45.1)

### Attendance

The majority of participants (76.4%) attended at least half of the sessions, with 22 (43.1%) participants attending 50–79% of sessions and 26 (16.6%) participants attending > 80% of sessions.

### Satisfaction

Fifty out of the 51 individuals in the analytical sample completed the mid-series satisfaction questionnaire. Table 2 shows results of satisfaction ratings for 11 areas. With the exception of one topic area (technical assistance from Project ECHO staff), over 90% of participants indicated that they were very satisfied/satisfied with each of the noted satisfaction areas. About a quarter (22%) of participants noted that technical assistance from Project ECHO staff was not applicable and therefore could not provide a satisfaction rating. Nonetheless, while participants found the intervention favorable overall, 1 out of 10 participants was dissatisfied/very dissatisfied with the duration of sessions, 8% were dissatisfied/very dissatisfied with engagement with other participants, and 6% were dissatisfied/very dissatisfied with the frequency of the sessions.

**Table 2:** Results from mid-series satisfaction survey (*n* = 50)

Topic area	Very satisfied/satisfied (%)	Dissatisfied/very dissatisfied (%)	Not applicable (%)
Registration process	92	0	8
Presentations (didactics)	100	0	0
Recommendations during case presentations	96	4	0
Engagement with other participants	88	8	4
Overall satisfaction with faculty	98	2	0
Curriculum and topics	98	2	0
Duration of sessions	90	10	0
Frequency of sessions	94	6	0
Technical assistance from Project ECHO staff	78	0	22
Communication with Project ECHO staff	94	0	6
Overall impression of Project ECHO for CHWs	98	0	2

### Changes in knowledge

Table 3 shows results from the pre- and post-knowledge poll questions from didactic sessions 5 through 7, and 9 through 12 (seven didactic sessions total). The knowledge poll data captured during the 8th session were invalid due to a technical glitch which resulted in the wrong pre-question presented during the didactic session. Overall, there was an average of 10% improvement in participants’ knowledge.

**Table 3:** Changes in participants’ knowledge (the knowledge poll data captured during the 8th session were invalid due to a technical glitch which resulted in the wrong pre-question being presented during the didactic session)

Session	Pre (% correct)	Post (% correct)	Percentage change (%)
Session 5	100	99	− 1.00
Session 6	90	100	+ 11.11
Session 7	70	88	+ 25.71
Session 9	90	81	− 10.00
Session 10	80	85	6.25
Session 11	92	96	4.35
Session 12	78	89	14.1

### Changes in self-efficacy

A paired sample *t* test was performed to compare self-efficacy scores among the 51 participants before and after the ECHO CHW initiative (Table 4). There was a statistically significant difference of + 0.453 when comparing self-efficacy scores before the initiative (M = 3.14, SD = .56) and after the initiative (M = 3.60, SD = .35; *t*(50) = 6.512, *p* < .001). The latent change score model calculation revealed a dose-response relationship between number of ECHO CHW sessions attended and improvements in self-efficacy. In other words, those who attended more ECHO CHW sessions showed a larger increase in self-efficacy. More specifically, for every 1 additional session attended, there was a .05 improvement in self-efficacy (or rather, 5 more sessions attended led to .25 larger change in self-efficacy). The latent change score model accounted for missing data due to attrition in survey completion pre- to post-engagement in ECHO CHW. A total of 119 participants completed a baseline, pre-series Enrollment Form; however, 51 completed the post-series survey. As such, we calculated a latent change score to create true change values in self-efficacy from pre to post without ignoring missing data at the post-series marker.

**Table 4:** Changes in self-efficacy (statistical significance detected if *p* < .05)

	Beta coefficient	Standard error (SE)	***p*** value
**Observed change**			
*n* = 51	.010	.024	0.67
**True change**			
*n* = 121	0.53	.009	0.00

### Behavior change intent

With regard to the three questions on behavior change intent that were included in the mid-series survey, three out of five (63%) participants endorsed the intention of approaching their work differently based on knowledge gained from Project ECHO, and four out of five (80%) participants indicated that they did not foresee any barriers to implementing the intended behavior changes. Over half (55%) of the participants, however, noted that they had not yet made any changes based on knowledge gained from Project ECHO.

### Participants’ perspectives on the program

Four major themes emerged from review of the qualitative data (Table 5). Two themes related to the usefulness of the ECHO CHW intervention, and two related to CHWs in the context of the healthcare team.

**Table 5:** Themes identified through qualitative analysis of participant interviews

Theme	Sample quotes
Intervention as a viable education tool for supporting CHW programming	*I'll have to say it (ECHO) was just more engaging. Most of my (other) trainings, I have to drive somewhere so it's really nice that I was sitting in my office. I didn't have to go anywhere. I really loved hearing all the cases and how people deal with all these different patients. Because they sound very similar to some of the patients that we all share.*
	*Sometimes, it's a little frustrating when you have a patient where you're more invested in getting things done than they are. And those (ECHO sessions) were good for me because I just needed some more-- I guess I needed my own motivation.*
	*I think it definitely has shaped the community health worker role here since we are just starting and forming that position. It's given us some guidelines to go by and some expectations that we haven't previously had.*
	*After working with the Project ECHO, it was very helpful and more hands-on for us because the ideas that other facilities gave us with ways to work with our patient helped us out a lot because we learned how other facilities work with their patients.*
	*It was helpful because we were able to start thinking about different situations that we hadn't seen, yet, hear other centers. So we were able to take that knowledge and keep it in mind for future situations.*
Heightened intent to improve patient-centered and team-based care practices	*I plan to focus more on educating patients and also on determining individual patient needs. By focusing on individual needs, I can increase the chances of success of the patient.*
	*I will work more with the patient so they can feel like we are both doing together instead of me doing it for them.*
	*Going forward I will utilize all (or more) members of my patient's care team to better serve our population.*
	*I look forward to sharing the tools and case study recommendations gained with my program teams and clinic leadership teams, in order to optimize on our current work and provide opportunities for supporting care teams working with patients in need of support (to help remove barriers and reach health goals).*
Variability in roles and responsibilities of CHWs	*One thing that's always kind of amazing to me, and just talking to community health workers around the state or to being part of an ECHO, is the difference that the agency chooses to utilize community health workers. So you do have those agencies that it's like a one-time, here's the issue, fix it, and you're done kind of thing. Whereas, for myself, I might get a referral. And I can get referrals from social workers or I can get them from therapists or nurses or I can get them from doctors.*
	*I know that some community health workers might only deal with diabetes. Other people deal all across the board with everything imaginable.*
	*We (CHWs at participating site) all have very different aspects and roles that we take care of. We have two CHWs that handle diabetes and we do OB. Then we have one CHW that just does transportation.*
Challenges in validating CHW’s added value to care team	*My main topic that I found that it was helpful through the ECHO was having to integrate yourself with the team. It's very difficult to let the team know that you are needed as a CHW. It is very difficult to work at a big health center and be able to gain the trust of other team members as providers and nurses working along with the patients, and let them know that you are here to help the patient as much as they are.*
	*Sometimes, we CHWs help the patient much more than the providers due to time limit. They have such a small time to be with a patient that the patient end up coming to us and talking to us of their problems and needs, and even medical problems. Sometimes, it's a challenge to let the provider know that the patient has talked to you on such intimate topics as their health and be able to relay that to them at the same time as not crossing the boundary, or that respect for the patient that they give you as talking to you about their problems.*
	*In the past, I don't think CHWs have been valued because it's not a service that you can get payment or reimbursement for, But, I think that people are open to the idea now of CHWs. It's like a new buzzword. People are picking up on it and so the support is growing. I think in the past, a lot of times people across the board have not valued nonclinical people, but I think that CHWs have a lot to offer whether they're clinical or not.*

*Themes related to the usefulness of the ECHO CHW intervention.* Two themes related to the usefulness of the ECHO CHW intervention that were constructed from the responses included *the intervention as a viable education tool for supporting CHW programming* and *heightened intent to improve patient-centered and team-based care practices*. Participants endorsed ECHO CHW as convenient due to the use of the video conference platform. Moreover, participants described feeling supported by being able to connect with peers from practices across the country who have shared challenges while learning about cases that they had not encountered before. Additionally, participants noted the program heightened their intent to improve patient-centered and team-based care practices, including feeling better positioned to engage patients in shared decision-making. Participants also noted their intent to use skills learned from the sessions to better support, leverage, and improve competencies of colleagues.

*Themes related to CHWs in the context of the healthcare team.* Two themes related to CHWs in the context of the healthcare team that were observed from participants’ responses included *variability in roles and responsibilities of CHWs* and *challenges in validating CHW’s added value to care team*. Several participants noted great variation in the responsibilities of CHWs within and across practice sites. For example, some practices engaged CHWs in screening and referral for health-related social needs (e.g., transportation), whereas others had CHWs more involved with chronic disease management. Nonetheless, some participants reported having a difficult time gaining the trust of colleagues due to reservations regarding CHWs’ added value and ability to assist with patients’ health and social needs. One participant attributed these challenges to policy-related factors, including lack of financial incentives and reimbursement structures to support inclusion of CHWs as part of healthcare teams.

Triangulation of data from both the pre-post surveys and focus groups demonstrated alignment regarding faculty satisfaction as a measure of acceptability, with 98% of the participants rating their satisfaction with faculty favorably. Several participants mentioned the importance of having a senior CHW as part of the faculty, suggesting that a senior CHW provides an opportunity for participants to learn from experienced professionals who have “traveled a similar road” and can advise on best practices. Additionally, both quantitative and qualitative findings suggest that participants’ self-efficacy significantly increased as a result of participation. Those with higher levels of engagement (as measured by number of sessions attended) experienced greater increases in self-efficacy from pre- to post-intervention. Focus group participants also shared that they had already modified their behavior based on information learned from the ECHO program or planned to do so in the future.

Nonetheless, not all aspects of the program were viewed favorably. Four out of five participants (80%) were dissatisfied or very dissatisfied with the ability to engage with other participants during the ECHO sessions. Focus group findings corroborated this finding. One participant shared that having other participants’ contact information would enhance interaction by enabling participants to communicate directly with each other during the sessions.

## Discussion and conclusion

This evaluation suggests that Project ECHO can be a useful tool to provide continuing education and training for CHWs. CHWs are increasingly being assigned to healthcare teams to help improve patient outcomes based on an increasing recognition of the critical role played by SDOH. These issues, while traditionally not emphasized in medical care, may be the key to reducing health inequality and improving the health and well-being of the broader population. Moreover, the challenge some participants faced gaining trust and validation of the importance of their role from the care team suggests that additional work is needed to educate front line clinical providers on the impact of SDOH and the need to address them as part of clinical care.

Through the use of mixed quantitative and qualitative methods, ECHO CHW participants provided robust perspective about the feasibility, acceptability, and impact of the initiative. Participants shared how certain skills and knowledge obtained from ECHO CHW could be deployed in their respective communities and healthcare settings. Knowledge and self-efficacy gains in didactic areas such as integrating trauma-informed care, enacting motivational interviewing skills, and changing patients’ health behaviors indicated its impact. The participants’ positive regard of the curriculum topics, didactic presentations, and case recommendations demonstrated acceptability. In acknowledging that the video conferencing environment was conducive to their learning needs, ECHO CHW participants provided context for the initiative’s overall feasibility.

Through attendance tracking and qualitative interviews with ECHO CHW participants, we learned about the convenience of connecting learners through the ECHO CHW video conferencing platform. This accessibility lent well to the feasibility of the pilot program. Participants spanning a wide geographic area connected with peers in order to share how they resolve SDOH-related issues in their patients’ lives. The project team anticipated that many of those who registered to participate in ECHO CHW would continue throughout the duration of the pilot program. Data show that of the 114 participants who remained actively enrolled in ECHO CHW, 8 participants (7.0%) did not attend any sessions as compared to 66 (54.4%) who attended at least 50% of the ECHO CHW sessions. While not conclusive, these attendance rates in addition to participants’ qualitative responses about how they would deploy content learned from ECHO help us better understand how feasible it was for participants to attend sessions of interest to them.

This study was unique in two important ways. First, the ECHO intervention was not focused on a specific disease or condition. Rather, this intervention provided continuing education and training more broadly and used cases to support and reinforce role-specific core competencies rather than to provide upskilling in a more specialized area. Though initially developed to improve access to evidence-based treatment for hepatitis C (Arora, Kalishman, & Thornton, [25]), subsequent ECHO programs have addressed other conditions such as substance abuse, pain care, diabetes, and hypertension ( [26]; Bouchonville, Hagar, Kirk, Qualls, & Arora, [18]; Katzman, Comerci, & Landen, [27]; Katzman, Fore, & Bhatt, [28]; Khatri, Haddad, & Anderson, [29]; Komaromy, Duhigg, & Metcalf A, [30]). While evidence is growing to suggest that disease-focused ECHO programs improve clinical outcomes (Anderson, Zlateva, Davis & Spegman, [31]; Bouchonville, Hagar, Kirk, Qualls, & Arora, [18], Kawasaki, [32]; Komaromy, Bartlett, Manis, & Arora, [33]; Korthuis, McCarty, & Weimer, [34]; Thies, Anderson, & Beals-Reid, [35]), less is known about the model’s efficacy to provide broader training not focused on a particular illness. Similarly, although one prior study evaluated the use of Project ECHO to train CHWs, it was focused specifically on diabetes (Zurawski, Komaromy, Ceballos, McAuley, & Arora, [17]).

Secondly, this intervention was unique in that the intended audience was comprised of CHWs. Most ECHO programs have been designed with medical providers in mind; however, less is known about the impact and utility of ECHO for other members of the healthcare team. In this study, CHWs were actively engaged participants. In addition to their high degree of satisfaction, participants demonstrated significant improvements in knowledge and behavior change intent, suggesting that participants, as they reviewed and discussed CHW core competencies during the didactics, were gaining confidence building skills that they would apply to their work.

Nonetheless, this study has several notable limitations. While we demonstrated increase in knowledge for some sessions, the overall response rate for completing the pre-post knowledge assessment was low (42%). The pre-session knowledge poll question was administered at the outset of each ECHO session. Unfortunately, not all attendees logged in on time, leading to low rates of survey completion. Moreover, knowledge assessment was limited to what was taught in the didactics part of each session. A broader pre-post knowledge assessment of CHW skills would have provided a useful addition to the topic-specific assessments conducted at each session. Lastly, while we demonstrated an increase in intent to apply new knowledge, additional studies are needed to more definitively assess whether these preliminary positive findings translate into measurable improvements in patient outcomes.

With these limitations in mind, we conclude that the Project ECHO model can play an important role in supporting and providing CHWs with continuing education to help address challenges faced by patients beyond the traditional domain of healthcare provision. Programs like ours that train and support CHWs are increasingly important as health systems, prompted in large measure by changes in payment models, seek new strategies to improve quality and value. Value-based payment models, with their emphasis on non-visit-related reimbursement, provide a powerful incentive for health systems to invest in interventions aimed at improving not only clinical process measures within the care delivery system, but larger, population-based measures as well. With increasing recognition of the impact of environmental and other factors on health outcomes, interventions such as CHWs can play an essential role in achieving clinical and financial goals for primary care practices and larger health systems. Project ECHO is a scalable tool with minimal technology requirements that nearly anyone in any location can access. Moreover, increasing recognition of the importance of CHWs has led many states to establish CHW certification requirements (London, Carey, & Russell, [36]) including requirements for continuing education. Programs such as ours can support CHWs in meeting these requirements and standardize training content across large geographic areas. Reducing health inequality will require a concerted emphasis on addressing SDOH, which CHWs are ideally suited to support. Project ECHO for CHWs can help ensure that this much-needed role is supported with evidence-based guidance and peer support and interaction.

## Supplementary information

**Additional file 1:.** Appendices A-D.

## Data Availability

The datasets used and/or analyzed during the current study are available from the corresponding author on reasonable request.

## Abbreviations

CHW: Community health worker
ECHO: Extension for Community Healthcare Outcomes
FQHC: Federally Qualified Health Center
SDOH: Social determinants of health
SIM: State Innovation Model
