# Supplementary material for: A mixed methods evaluation of the feasibility, acceptability, and impact of a pilot project ECHO for community health workers (CHWs)
Source: Pilot Feasibility Stud. 2020 Sep 18;6:132. doi: 10.1186/s40814-020-00678-y (PMC7499981; doi:10.1186/s40814-020-00678-y)

**Appendix A. Moore’s Model of Outcomes Assessment**

|  |  |  |  |  |  |  |
| --- | --- | --- | --- | --- | --- | --- |
| **Level** |  | **Framework** |  | **Description** |  | **Potential Data Sources** |
| 1 |  | Participation |  | The number of persons who participated in the activity |  | Attendance Records |
| 2 |  | Satisfaction |  | Degree to which expectations about setting and delivery were met |  | Post-activity questionnaire |
| 3A |  | Learning: Declarative Knowledge |  | Degree to which participants state what the activity intended them to know |  | Pre- and post-tests of knowledge; self-report of knowledge gain |
| 3B |  | Learning: Procedural Knowledge |  | Degree to which participants state how to do what the activity intended them to know how to do |  | Pre- and post-tests of knowledge; self-report of knowledge gain |
| 4 |  | Competence |  | Degree to which participants in an educational setting demonstrate how to do what the activity intended them to be able to do |  | Observation in educational setting; self-report of competence; intention to change |
| 5 |  | Performance |  | Degree to which participants can do what the activity intended them to be able to do in their practices |  | Observation of performance in patient care setting; patient charts; administrative databases; self-report of performance |
| 6 |  | Patient Health |  | Degree to which the health status of patients improves due to changes in participants' practice behavior |  | Health status measure recorded in patient charts or administrative databases; patient self-report of health status |
| 7 |  | Community Health |  | Degree to which the health status of a community of patients changes due to changes in participants' practice behavior |  | Epidemiological data and reports; community self-report |

**Appendix B.** ECHO CHW Intervention Didactic Topics.

| **Topic** | **Objectives** |
| --- | --- |
| Qualitative Interviewing Skills: Understanding Root Causes | Interviewing and understanding patients as people  - Listening skills  - Identifying the root causes of health troubles |
| CHWs as Members of the Care Team | Care team integration  - How to help patients get the most out of their appointments  - Guiding patients through the clinic appointment  - Practicing talking to doctors |
| Ending the Patient Relationship | Making a good ending for patients and for CHWs  - Reasons for ending the patient relationship  - Strategies for ending the patient relationship  - Connecting to other sources of support |
| Trauma-Informed Care | How trauma affects patients  - Ways in which experiences of trauma affect patient care  - Trauma and substance use  - Skills CHWs can use to be trauma-informed |
| Changing Health Behavior | Tools to understand how beliefs, attitudes, norms, and barriers impact behavior  - Skills CHWs can use to support patients in changing behavior  - Practicing CHW self-awareness and identifying biases |
| Re-engaging Patients | Working with patients who lose touch or lose motivation  - Creative strategies for re-engaging patients  - Skills to help patients stay motivated toward their goals |
| Responding to Emergency Situations | Responding to urgent patient-related challenges  - Medical, psychiatric, substance use and domestic violence crises  - How to respond to and resolve crises as a CHW |
| Ethics, Patient Privacy, and Boundaries | CHW Code of Ethics  - Patient privacy  - Navigating tricky ethical situations and professional boundaries |
| Connecting Patients to Resources | Making meaningful and useful connections  - How to make good referrals for patients  - Tips for finding creative, out-of-the-box resources  Tools for locating new resources in your community |
| Supporting Patients with Mental Health Concerns | Mental health challenges patients face  - Ways in which mental health challenges affect patient care  - Skills CHWs can use to support patients with mental health concerns |
| Re-Directing Patients | Working with patients who cross boundaries  - Re-directing patients and maintaining boundaries |
| Conflict Resolution & De-escalation | Responding to and resolving conflict as a CHW  - Conflict resolution and de-escalation tips and techniques |

**Appendix C. ECHO CHW Intervention Focus Group Questions.**

*Focus Group Questions Part 1 (45 minutes)*

**Part 1: Content**

1. What new skills or knowledge were you expecting to gain from this Project ECHO series?
2. How would you compare your experience in ECHO CHW with other types of support offered to you?
3. What didactic topic(s) did you find most helpful?
4. What are your recommendations for improving the content?

**Part 2: Attendance**

1. Were there ever times when you planned to attend a session, but couldn’t do so? If so, what was the main barrier?
2. How satisfied were you with the length of the didactics and case presentations?

**Part 3: Resources**

1. What resources did you expect to access during this series?
2. What are your suggestions for additional resources the team could have provided to complement learning?

**Part 3: Engagement & Application**

1. To what extent, if at all, has this intervention changed the way you engage in your professional role?
2. To what extent do you apply knowledge gained in ECHO CHW to your professional role?

[Case Presentations]

- How beneficial did you find the case presentations to your work?
- If you presented a case, what was your experience like? What recommendations would you suggest to make it any different?

**Part 5: Support & Relationships**

1. How would you describe the support you receive from your health center to apply the skills or tools you learned in this series?
2. How would you describe your relationship with your patients/clients since participating in the series?
3. To what extent, if at all, has your relationship with clinical staff (primary care, behavioral health, dental, etc.) changed since participating in ECHO CHW?

**Part 7: Closing**

1. Is there anything you’d like to share that we haven’t discussed?

*Closing (2 minutes)*

Thanks for joining today and sharing your opinions. Your comments have given us lots of different ways to assess *Project ECHO for Community Health Workers*. Thank you for your time, and enjoy the rest of your day.

**Appendix D. States Participating in ECHO Community Health Workers Intervention**


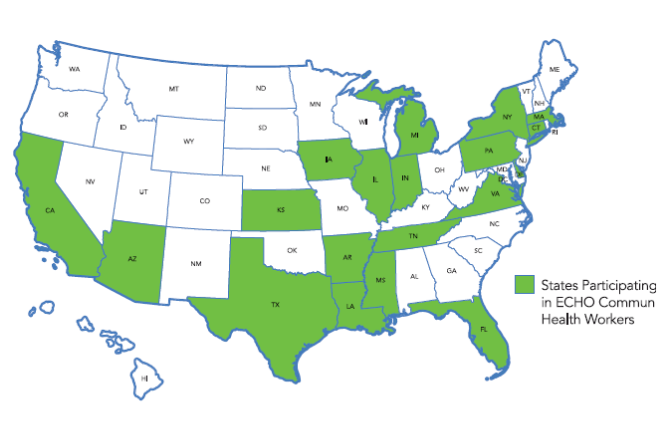

Supplement: Supplementary file 1 — Additional file 1:. Appendices A-D. [file 40814_2020_678_MOESM1_ESM.docx]
